# Layered Enzymatic Biosensor Decorated with Prussian Blue Structures for Continuous Electrochemical Glucose Sensing

**DOI:** 10.1021/acsomega.5c08879

**Published:** 2026-04-16

**Authors:** Aline Macedo Faria, Glauco Meireles Mascarenhas Morandi Lustosa, Talita Mazon

**Affiliations:** Centro de Tecnologia da Informação Renato Archer, Ministério da Ciência, Tecnologia e Inovação (MCTI), Dom Pedro I Highway, Km 143.6, 13069-901 Campinas, São Paulo, Brazil

## Abstract

Continuous glucose monitoring is a pressing need in the management of diabetes mellitus. In this study, we report the development of an innovative electrochemical enzymatic biosensor engineered by immobilizing glucose oxidase on a cystamine-modified ENIG (electroless nickel immersion gold) electrode on a printed circuit board and decorating it with spherical Prussian Blue nanostructures with diameters of 30–90 nm. This multilayered architecture demonstrated enhanced electron transfer efficiency and stable chronoamperometric responses across varying glucose concentrations. The biosensor exhibited immediate, reproducible current changes upon glucose addition, with superior sensitivity at an optimized Prussian Blue concentration of 5 mM. Stability tests conducted over 8 days confirmed consistent performance, but highlighted the need for postanalysis regeneration steps. Furthermore, real-time monitoring experiments in a simulated interstitial fluid environment demonstrated the sensor’s ability to maintain functionality over extended immersion periods, delivering rapid, distinct electrochemical signals in response to glucose fluctuations. These results underscore the potential of this biosensor design for future applications in minimally invasive, continuous glucose monitoring systems.

## Graphical abstract

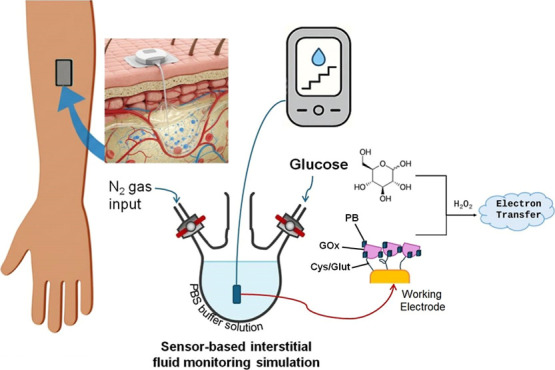

## 1 Introduction

Diabetes mellitus is a chronic metabolic disorder affecting millions of people worldwide, with projections indicating up to 780 million cases by 2045.^1–4^ This metabolic disorder disrupts the body’s ability to regulate blood glucose levels, leading to severe long-term complications such as retinopathy, nephropathy, cardiovascular diseases, and diabetic foot syndrome, which is characterized by neuropathic, ischemic ulcers and an increased risk of mortality.^4,5^ Because glucose levels fluctuate significantly due to diet, physical activity, stress, and metabolic variability, continuous and reliable monitoring is essential. Clinical reference values include: (i) normal fasting glucose levels (3.9–6.1 mmol/L, 70–110 mg/dL), (ii) impaired fasting glucose or prediabetes (6.1–6.9 mmol/L, 110–125 mg/dL), and (iii) diabetes: (≥7.0 mmol/L, 126 mg/dL).^6^ Continuous monitoring and appropriate glycemic management are therefore crucial to maintaining quality of life and reducing the socioeconomic burden associated with diabetes.^7,8^

Effective diabetes management depends on accurate and frequent glucose measurements, as glycemic variability is strongly associated with acute events, such as hypoglycemia and long-term complications, including retinopathy, nephropathy, and cardiovascular disease. Traditional finger-prick self-monitoring of blood glucose (SMBG), although widely adopted, is invasive, discontinuous, and often associated with poor long-term adherence.^9^ Continuous glucose monitoring (CGM) has emerged as a transformative alternative, offering real-time glucose trends, improved time-in-range, and reduced asymptomatic hypoglycemic episodes.^10^ Clinical and economic studies demonstrate that CGM contributed to lower HbA1c levels, higher patient satisfaction, and reduced healthcare utilization in both type 1 and type 2 diabetes.^11–13^

Electrochemical glucose sensing technologies have evolved substantially over the past few decades. Early devices relied on disposable enzymatic strips coupled to portable potentiostats, enabling single-point measurements through capillary blood sampling.^14^ Although these systems revolutionized diabetes care, they are inherently invasive and unsuitable for continuous metabolic tracking. More recently, CGM platforms employed miniaturized electrochemical probes that operate in interstitial fluid (ISF), which has emerged as the most reliable biological matrix due to its strong correlation with blood glucose levels and greater physiological stability than saliva, sweat, or tears.^15^ However, many reported enzymatic glucose sensors remain limited to short-term laboratory measurements, complex nanostructured fabrication routes, or lack validation under continuous immersion conditions that realistically simulate CGM operation.^16,17^

In the earliest generations of enzymatic glucose sensors, glucose oxidase (GOx) was typically immobilized via physical adsorption or by entrapment within polymeric matrices. While these strategies enabled the first practical sensors, they suffered from weak enzyme retention, random orientation, and limited long-term stability, particularly under prolonged or continuous use.^18,19^ Although effective, these configurations rely on proprietary materials, multilayer polymer engineering, and sophisticated microfabrication processes, increasing production cost and limiting scalability and flexibility.^19–21^

Electrochemical glucose sensors are generally classified as nonenzymatic or enzymatic. Nonenzymatic systems rely on metal oxides or nanostructures to directly catalyze glucose oxidation,^22–25^ but often exhibit poor selectivity in complex biological environments. Significant efforts have therefore been directed toward the development of electrochemical glucose sensors,^26–30^ owing to their high sensitivity, selectivity, rapid response, low cost, and reliability.^5,31^ Enzymatic sensors, based on Gox, remain dominant due to their high specificity and reliability.^32–35^ In these systems, Prussian Blue (PB, ferric hexacyanoferrate) has been extensively used as an electrocatalytic mediator for hydrogen peroxide reduction, enabling low-potential operation (∼0.0 V vs Ag/AgCl) and minimizing interference^36–44^ However, PB suffers from limited operational stability, as hydroxide ions generated during H_2_O_2_ reduction can promote its dissolution.^45,46^ Numerous stabilization strategies, such as nanostructured supports, composite films, codeposition with other hexacyanoferrates, or polymer embedding have been proposed.^45,47–50^ Although effective, these methods often involve multistep electrodeposition or complex nanomaterial fabrication, reducing compatibility with scalable microelectrode and PCB-based platforms and limiting practical translation of CGM.^16,17^

Despite the extensive literature on GOx-PB systems,^51–56^ important challenges remain unresolved. Many reported sensor architectures depend^51,55,56^ on multilayer Langmuir–Blodgett (LB) film assemblies, complex nanostructured supports, or multistep electrodeposition strategies to improve the stability of PB. Although these approaches can enhance electrocatalytic performance, they often involve limited scalability. Moreover, a significant portion of published studies emphasize static measurements or short-term analytical performance, rather than true continuous monitoring under physiologically relevant conditions.^56^ The intrinsic instability of PB during prolonged operation due to hydroxide-induced dissolution in neutral or slightly alkaline media remains a critical barrier to its reliable integration into CGM devices.^56^ Several stabilization strategies have been proposed, including composite film formation, codeposition with metal hexacyanoferrates, polymer encapsulation, and incorporation into nanostructured scaffolds.^56–60^ While effective to some extent, these solutions frequently increase fabrication complexity, introduce additional interfacial resistance, and reduce compatibility with scalable microelectrode and printed circuit board (PCB)-based platforms.^52^ Therefore, developing low-cost, structurally simple electrochemical biosensors based on straightforward PB deposition and controlled GOx immobilization represents an important step forward. Such an approach aims to balance electrode stability, enzyme activity, and continuous signal generation during long-term operation, paving the way for next-generation CGM devices.

In this context, the present work introduces a simplified yet chemically robust multilayer architecture for continuous glucose monitoring, implemented on an ENIG-finished PCB sensor platform. The proposed system departs from traditional thick polymeric matrices and complex nanostructured assemblies. It employs a rational surface chemistry strategy based on a cystamine self-assembled monolayer (SAM) on gold, followed by the glutaraldehyde-mediated covalent immobilization of GOx.

Specifically, a cystamine-based SAM was applied as a prefunctionalization layer on the ENIG-finished electrode. Then, glutaraldehyde was mixed with the GOx solution prior to deposition on the modified electrode. This dual modification approach enhanced both structural and functional performance, promoting uniform deposition of PB nanoparticles (30 and 90 nm), enhancing enzyme immobilization, and ensuring stable electron-transfer pathways. As a result, the reproducibility and stability were improved.

Importantly, unlike earlier SAM/LB-based ultrathin biosensors, typically evaluated under static conditions, the present study emphasizes validation under continuous operation, more closely reflecting real-world CGM demands. While many reported systems use complex microneedle coatings, such as polydopamine (PDA), reduced graphene oxide (rGO), or platinum nanoparticles (Pt NPs),^51^ the present approach prioritizes structural simplicity, PCB compatibility, and stable long-term electrochemical performance.

Furthermore, the biosensor was systematically evaluated under prolonged immersion in simulated interstitial fluid conditions (PBS containing 70 mg/dL glucose), a scenario rarely explored in conventional PB-GOx systems.^61^ The device showed stable chronoamperometric (CA) responses over 8 days, with reproducible current variations upon glucose fluctuations and optimal sensitivity at a PB concentration of 5 mM. This continuous operational validation directly addresses a critical limitation of prior ENIG-SAM-GOx-PB architectures.

## 2 Experimental Section

### 2.1 Reagents and Materials

All chemicals used were of analytical grade. The following reagents were employed: phosphate-buffered saline (PBS, Laborclin), Silver ink (Ag/AgCl ink ALS), glutaraldehyde 50% (Alfa Aesar), cystamine dihydrochloride (Sigma-Aldrich), glucose oxidase (GOx) from *Aspergillus nigri* (Sigma-Aldrich), Flavin adenine dinucleotide (FAD) (Sigma-Aldrich), Prussian blue (Sigma-Aldrich), d-glucose (Sigma-Aldrich). All chemicals were of analytical purity and were used without further purification. All solutions were prepared with deionized water (resistivity of 18.2 MΩ).

### 2.2 Electrochemical Glucose Sensors Assembly

The multilayered enzymatic biosensor was fabricated using an integrated three-electrode sensor platform based on ENIG (electroless nickel immersion gold)-finished PCB. In this configuration, a chemically deposited nickel barrier layer, coated with a thin immersion gold layer, provides a flat, corrosion-resistant, and highly reproducible gold surface suitable for electrochemical measurements. The PCB-based sensor board was initially designed with three integrated ENIG electrodes, corresponding to the possible electrode layouts evaluated in this study, as illustrated in Figure S1. All electrodes were first fabricated in ENIG. Subsequently, the reference electrode (RE) was prepared by modifying one of the gold electrodes with a Ag/AgCl conductive ink. The RE was obtained by depositing Ag/AgCl conductive ink onto the selected ENIG electrode, followed by thermal curing at 100 °C for 15 min to promote solvent evaporation, improve adhesion, and ensure proper crystallization of the silver chloride phase. This approach represents a standard, strategically advantageous method for modern electrochemical biosensor fabrication. The Ag/AgCl system, one of the most stable and reproducible electrochemical redox couples, provides a well-defined and stable potential against which the working electrode (WE) is measured. In addition, it offers important practical advantages, including low cost, good electrical conductivity, and strong resistance to oxidation.^62,63^ The WE surface was then functionalized by forming a SAM of cystamine, which provides terminal amine groups for subsequent enzyme immobilization.

Initially, all electrodes were subjected to a rigorous cleaning protocol consisting of ultrasonic agitation in 3% Extran detergent, followed by thorough rinsing with deionized water (18.2 MΩ·cm) and air-drying at ambient conditions. This cycle was repeated three times to ensure the complete removal of surface contaminants and residues that could interfere with subsequent functionalization. For functionalization of the WE, 8 μL of a 20 mM cystamine dihydrochloride solution was dispensed onto the electrode surface and incubated at 100 °C for 10 min. This treatment generated a SAM that exposed terminal amine functionalities, serving as anchor sites for the subsequent covalent binding of biomolecules. Enzyme immobilization was performed by preparing a solution of glucose oxidase (GOx, 0.4 mg/mL) supplemented with flavin adenine dinucleotide (FAD, 0.2 mg/mL) to ensure the enzymatic activity. Glutaraldehyde was added to a final concentration of 2.5% (v/v) to promote Schiff-base cross-linking between the aldehyde groups and the primary amines of cystamine and GOx. An 8 μL aliquot of this mixture was then deposited on the cystamine-modified WE and dried at 37 °C to yield a stable enzyme layer.

To improve the electron transfer kinetics, Prussian Blue (PB) mediator solutions were prepared by ultrasonic agitation at ∼60 °C for 25 min. This process ensured complete dissolution and a homogeneous dispersion of the Fe­(II)/Fe­(III) hexacyanoferrate framework. PB was initially prepared in solutions at concentrations of 5, 10, and 20 mmol. Next, 8 μL of each PB solution was drop-cast onto the enzyme-coated WE and dried at 100 °C, forming the electrocatalytic layer. Comparative analyses showed that PB concentrations of 5 and 10 mM provided the most uniform distribution of nanostructures. This prevented agglomeration and enhanced catalytic activity toward hydrogen peroxide reduction, maximizing the sensor performance. Therefore, these concentrations were selected for further experiments. The final three-electrode configuration consisted of the multilayer PB/GOx/FAD/glutaraldehyde/cystamine/Au structure on the WE (as shown in Figure S1), an Ag/AgCl RE and ENIG CE. Together they enable precise electrochemical measurements.

To assess the reproducibility of the sensor response, measurements were performed by using independently fabricated sensors prepared in triplicate. In these new sensors, Prussian Blue (PB) aliquots were deposited at concentrations of 5 mmol (designated Sensor 1, Sensor 2, and Sensor 3) and 10 mmol (designated Sensor 4, Sensor 5, and Sensor 6). Furthermore, to evaluate repeatability and short-term stability, additional sensors were prepared using 10 mmol of PB (Sensor 7, Sensor 8, and Sensor 9) and electrochemically characterized over a period of 8 days.

### 2.3 Characterizations

Characterization Techniques Microscopic imaging of the WE surface was conducted using a Bioval optical microscope (model 2110494) equipped with 10× and 40× objectives to evaluate PB distribution, enabling visual inspection and documentation of the electrode surface prior to experimental procedures. The biosensor was also evaluated by field-emission gun scanning electron microscopy (FEG-SEM), performed on a Tescan model Mira 3 XMU operated at 5 kV for secondary electrons (SE). These microscopic images provided insights into structural characteristics from the homogeneous distribution of Prussian blue nanostructures on the electrode material.

Fourier-transform infrared spectroscopy (FTIR) was carried out within a wavelength range of 650–4000 cm^–1^ (resolution of 4 cm^–1^) in a PerkinElmer Spectrum 100 instrument to analyze the chemical composition and identify the functional groups (and their bonds) present in the material. This technique is based on the principle that molecules absorb infrared radiation at specific frequencies, corresponding to the vibration energies of their chemical bonds. When infrared radiation is applied to a sample, the molecules absorb part of this radiation, promoting vibrational transitions. The resulting spectrum is characteristic of each substance.

Electrochemical characterizations were performed by immersing the integrated three-electrode sensor in PBS (0.1 M, pH 7.4) at room temperature. The sensor was connected to a portable potentiostat (PalmSens, The Netherlands) controlled by PSTrace 5.9 software. CA measurements evaluated the analytical performance for glucose detection. Initially, a constant potential of +0.30 V (vs Ag/AgCl) was applied and an initial equilibration period of 60 s was allowed. Continuous current monitoring was followed for up to 600 s. Aliquots of d-glucose standard solutions (100, 200, 400, and 800 mM) were added at predetermined intervals. The resulting steady-state currents were recorded. The applied potential was selected to favor the electrocatalytic reduction of hydrogen peroxide mediated by Prussian Blue. This choice minimized background current and ensured both the sensitivity and stability of the amperometric response.

## 3 Results and Discussion

### 3.1 Biosensor Surface Characterization

To enhance the electrochemical performance of the as-prepared enzymatic biosensor, iron­(III) hexacyanoferrate (II) (Fe_7_N_18_C_18_), commonly known as Prussian Blue (PB), was employed as a redox mediator. Surface analysis and distribution of PB on the WE area were assessed by optical microscopy, as shown in Figure 1. Sensors fabricated without a cystamine prelayer exhibited nonuniform PB distribution and aggregate formation, which negatively impacted the electrochemical performance (Figure 1A). In contrast, electrodes treated with cystamine exhibited a more homogeneous PB layer, particularly at 5 and 10 mM concentrations. The formation of well-defined PB nanostructures was especially evident with 20 mM PB, but it also led to excessive film formation. This was not optimal for the sensor response (Figure 1B). Uniform and well-controlled PB deposition is essential to ensure continuous electron-transfer pathways and minimize current fluctuations. This improves signal stability and the reproducibility of the amperometric signal. In this context, electrodes modified with cystamine showed significantly more stable redox behavior and lower background noise. Poorly distributed or aggregated PB domains can cause discontinuities in the conductive network, leading to localized overpotentials, uneven current distribution, and progressive signal drift during repeated measurements. Cystamine plays a crucial role in modulating film formation. As a bifunctional molecule containing terminal amine groups, cystamine can improve surface adhesion and provide anchoring sites. These sites favor more uniform growth of the PB nanostructures.

**Figure 1:**
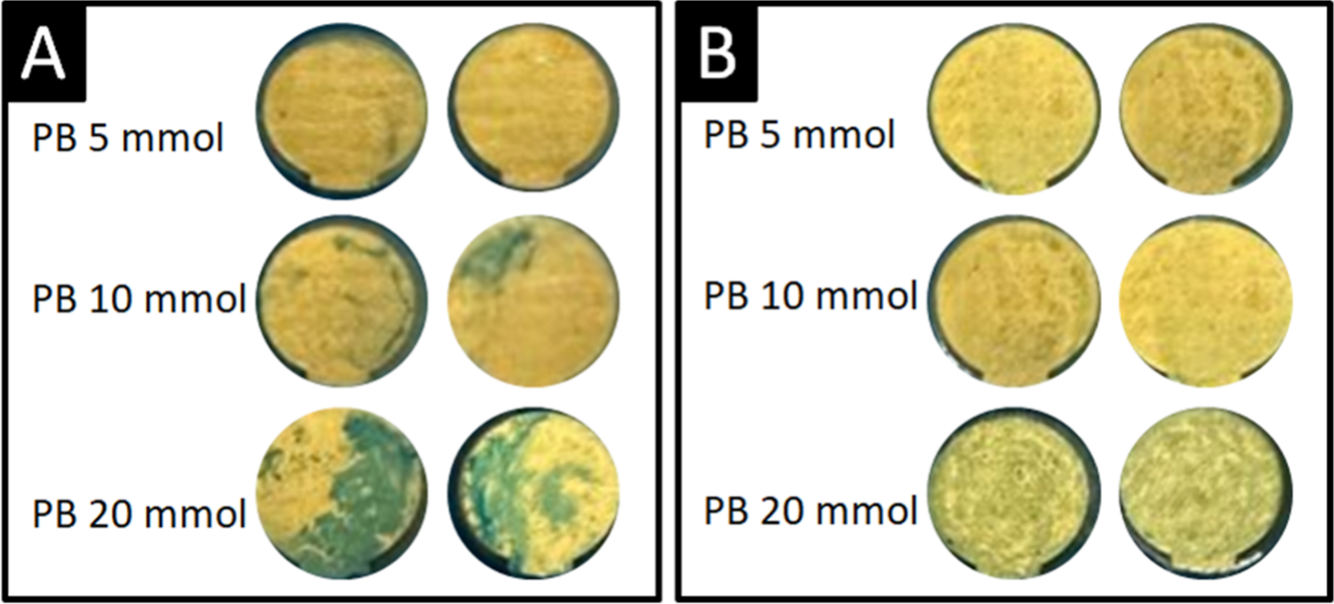
Comparative images between the WE area prepared (A) without and (B) with Cystamine and different concentrations of Prussian Blue (AP).

The PB nanostructures were characterized by SEM, as shown in Figure 2. The micrographs reveal a uniform and homogeneous distribution of the PB nanostructures across the WE surface. This uniformity is a key factor in sensor reliability as it ensures consistent electrochemical activity across the entire active area, preventing signal variability and enhancing reproducibility between measurements. The particle diameters were determined to be in the range from 30 nm up to 90 nm, as indicated in Figure 2E,F. This well-defined and nanoscale morphology provides a high surface area/volume ratio, which maximizes the number of active sites available for the electrochemical reaction and also can significantly accelerate electron transfer rates, thereby directly contributing to enhanced sensitivity and a more robust sensor signal.

**Figure 2:**
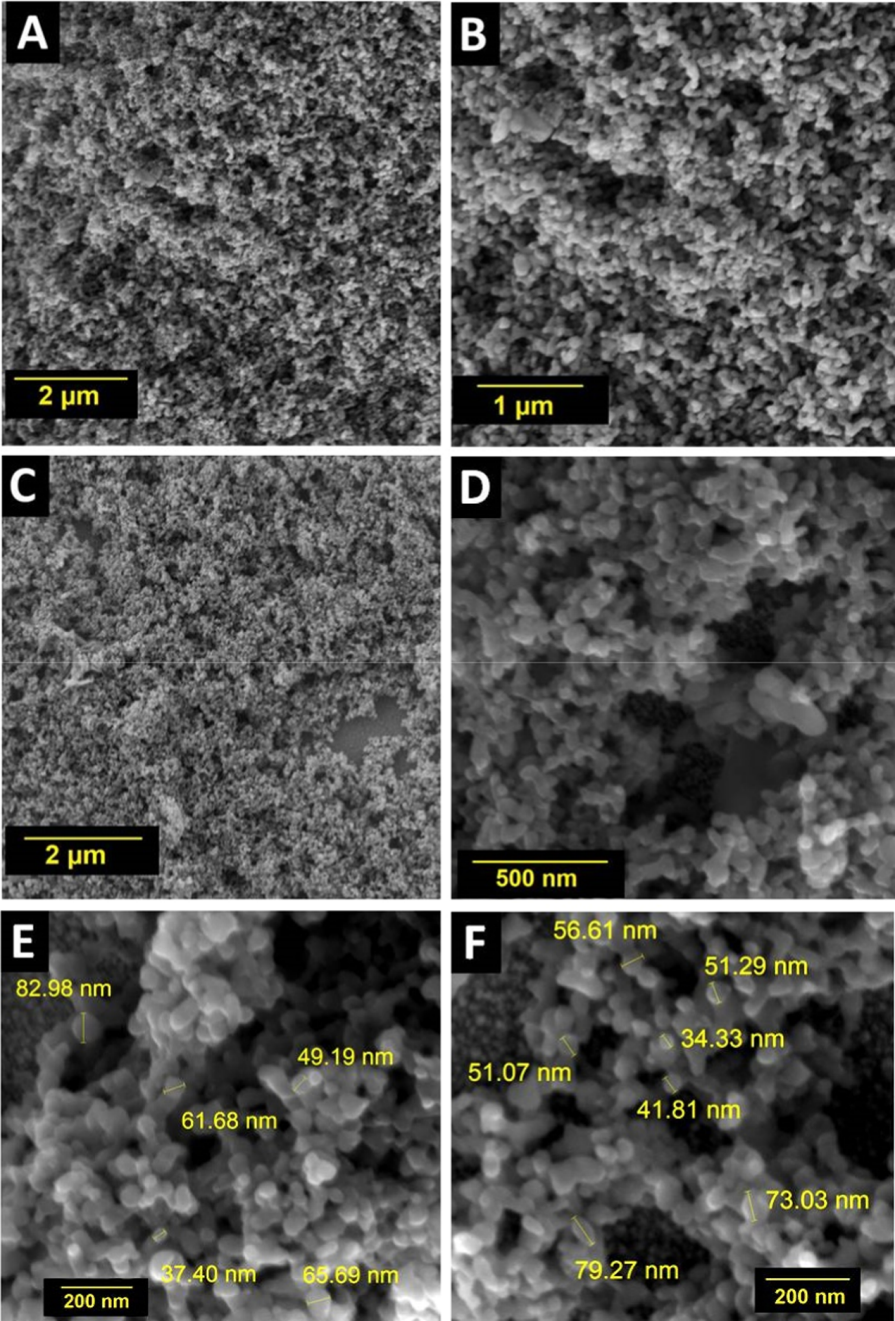
(A–F) SEM images of different regions from WEs with 5 mmol Prussian Blue nanoparticles.

The deposition of a cystamine prelayer onto the gold WE played a dual role in the overall biosensor architecture. First, cystamine molecules self-assembled as an amine-terminated monolayer, enabling subsequent covalent attachment of glucose oxidase (GOx) via glutaraldehyde cross-linking. This well-ordered interface improved the orientation and retention of the enzyme while reducing nonspecific adsorption on bare gold, thereby preserving catalytic activity under operational conditions.^64,65^ Importantly, the covalent immobilization pathway provided by the cystamine-glutaraldehyde system minimizes the conformational mobility of GOx, thereby mitigating denaturation and leaching during repeated electrochemical cycles.^66^ Second, the cystamine SAM exerted a marked influence on the deposition behavior of PB. Microscopy analyses demonstrated that electrodes lacking cystamine displayed heterogeneous PB aggregates and discontinuous coverage, whereas cystamine-modified electrodes exhibited a uniform and homogeneous PB distribution, particularly at the optimized 5 mM concentration. This behavior can be attributed to the ability of thiol-based SAMs to provide well-defined interaction sites, facilitating the ordered immobilization of PB nanostructures and stabilizing their electrocatalytic activity. Such effects are consistent with previous studies demonstrating that thiol SAMs promote controlled growth and stronger anchoring of PB nanoparticles on gold electrodes.^67^

The formation of the PB nanostructured layer is promoted by solvent-evaporation-induced crystallization. Upon deposition of the PB aliquot, evaporation of the solvent led to a state of supersaturation, leading to the growth of PB nanocrystals. The amine and thiol terminal groups likely facilitate the ordered anchoring of PB, directing the growth of nanostructures on the electrode surface and resulting in a more stable and electrochemically active surface. When it contacts the gold (Au) presented in the ENIG WE, the S–S bond from cystamine forms strong Au–S (thiolate) bonds, slightly protonating the amine groups of the molecule (–NH_3_^+^) and then attracting electrostatically the ferrocyanide group ${\left[{Fe\left(CN\right)}_{6}\right]}^{4-}$ from PB molecules.^68^ This molecular interfacial engineering not only enhances film stability but also may suppress mechanical detachment or structural degradation during prolonged electrochemical operation.

These findings align with prior reports on the role of thiol-based SAMs in directing the growth of nanostructured mediator^64^ and extend them by demonstrating a dual contribution of cystamine, thus facilitating both enzyme anchoring and mediator organization. To the best of our knowledge, this is among the first demonstrations of cystamine simultaneously enhancing enzymatic immobilization and regulating PB nucleation on PCBs, thereby providing a synergistic improvement in catalytic efficiency and operational reliability.^61^ In addition, the incorporation of glutaraldehyde during enzyme immobilization reinforced the stability of the biorecognition layer by forming Schiff-base covalent bonds between aldehyde groups and primary amines of cystamine and GOx. This three-dimensional cross-linked network effectively secured the enzyme at the electrode surface, reducing desorption during long-term testing. Such stabilization has been widely reported to extend the lifetime and robustness of enzyme-based biosensors,^65,66^ and in our study, it was essential to achieve consistent amperometric responses under continuous operation. Collectively, these results underscore that the synergistic combination of cystamine SAMs and glutaraldehyde cross-linking provides a robust and reproducible platform for enzyme immobilization and mediator integration in electrochemical biosensors.

Optimizing the PB concentration revealed that 5 mM yielded the most uniform and stable film, resulting in efficient electron transfer and reproducible amperometric responses. In contrast, electrodes modified with 10 or 20 mM PB showed irregular aggregated deposits, as confirmed by optical microscopy. Such thicker, heterogeneous PB layers are known to increase charge-transfer resistance, hinder mass transport, and reduce the accessibility of active catalytic sites, reducing sensitivity. This observation is consistent with previous studies emphasizing the importance of mediator loading in balancing catalytic efficiency and film stability in enzymatic biosensors.^69,70^

FTIR confirmed the successful stepwise functionalization of the electrode surface (Figure 3). Compared with the bare gold electrode (black line), the cystamine-modified surface (20 mM, red line) showed additional absorption bands that became more pronounced after GOx immobilization (green line). A characteristic band at 1613 cm^–1^ was assigned to the C≡O stretching of amide groups (–CONH_2_), consistent with the amide I region of proteins, confirming Schiff-base formation during cross-linking. Several bands associated with N–H stretching vibrations of amino groups from cystamine and the enzyme were also observed at 3360, 2965, and 2935 cm^–1^.^71^ Additional peaks were detected at 2837, 1441, 1318, 1270, 888, and 728 cm^–1^ (C–H stretching and bending modes) as well as at 1500 cm^–1^ (C–H vibrations) and within the 1400–860 cm^–1^ region (C–H angular deformations and C–N stretching). A distinct band at 1208 cm^–1^ was attributed to S≡O vibrations, indicative of cystamine on the surface. Finally, the FTIR spectrum of the biosensor displayed a characteristic band at ∼2105 cm^–1^, corresponding to the C≡N stretching of Fe–C≡N–Fe cyanide bridges, a fingerprint of Prussian Blue.^72,73^ Collectively, these spectral features validated the successful incorporation of cystamine, GOx, and PB into the biosensor structure.

**Figure 3:**
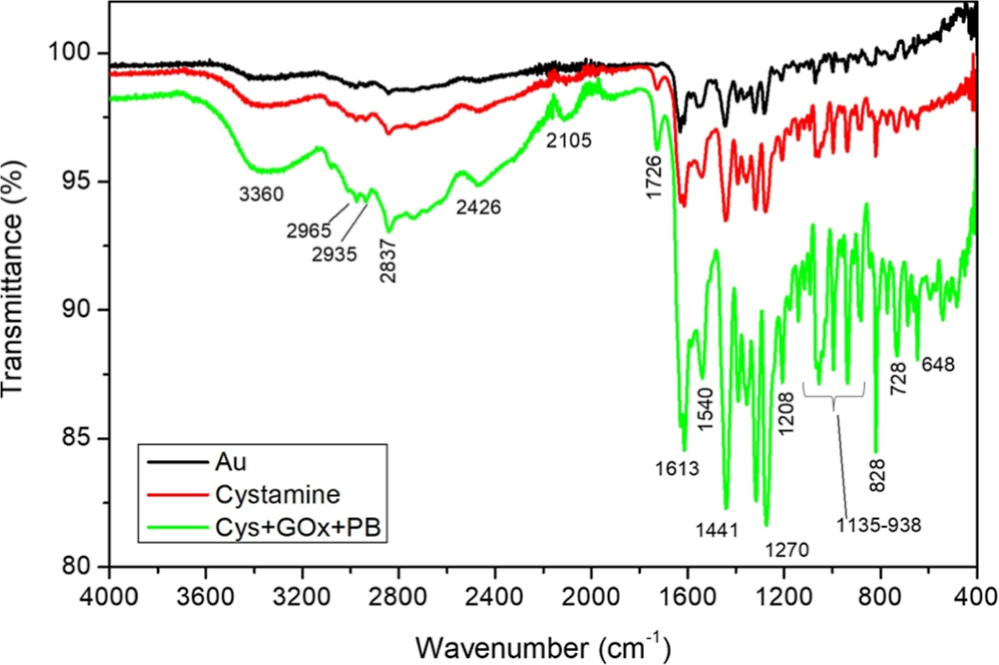
FTIR spectra of gold sensors (black line), sensor with 20 mM Cystamine (red line), and sensor with 20 mM Cystamine + GOx + Prussian Blue (green line).

### 3.2 CA Measurements

Preliminary studies tested equilibrium potentials of 100, 300, and 600 mV, while they tested at 100 mV. Figure 4 shows that using 100 mV produces a negligible signal, while 600 mV results in signal instability. By applying 300 mV a stable response was observed, which was adopted as the equilibrium voltage for all further analyses.

**Figure 4:**
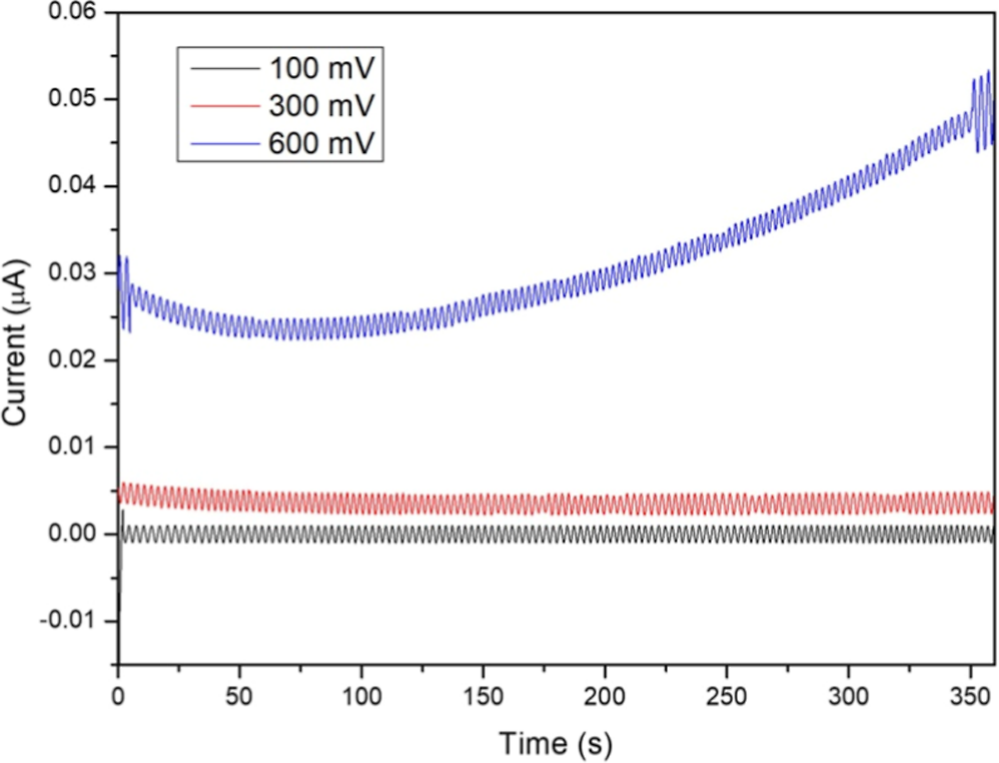
Comparative graphs of CA responses according to the applied potential to determine the equilibrium voltage.

The experiments determined the required volume of glucose solution for the electrochemical cell with an immersed biosensor. Sensor response was analyzed using four sequential glucose additions: 100, 200, 400, and 800 mM. A PBS-only control showed a slight increase in background current (∼200 to ∼360 μA) after 300 s, indicating steady-state diffusion effects. This is seen as the black line in Figure 5 (blank analysis). When 250 μL of a glucose solution was added (red line), no significant changes in the electrical response occurred. The red line rose linearly, similar to the blank analysis (black line), ranging from ∼150 to ∼320 μA. In comparison, the total variation in the electrical response for the red line curve was ∼170 μA, and for the black curve was ∼160 μA, which produced no significant deviation from the blank. Adding 500 μL of glucose (blue line), the sensor exhibited immediate (1–2 s) and reproducible current peaks. Successive glucose additions (100, 200, and 400 mmol) yielded consistent response peaks of 10.14 μA, 11.26 μA, and 10.20 μA, respectively. However, the 800 mmol addition resulted in a much higher peak of 32.76 μA. Based on these results, a 500 μL aliquot was standardized for further experiments to ensure a detectable sensor response.

**Figure 5:**
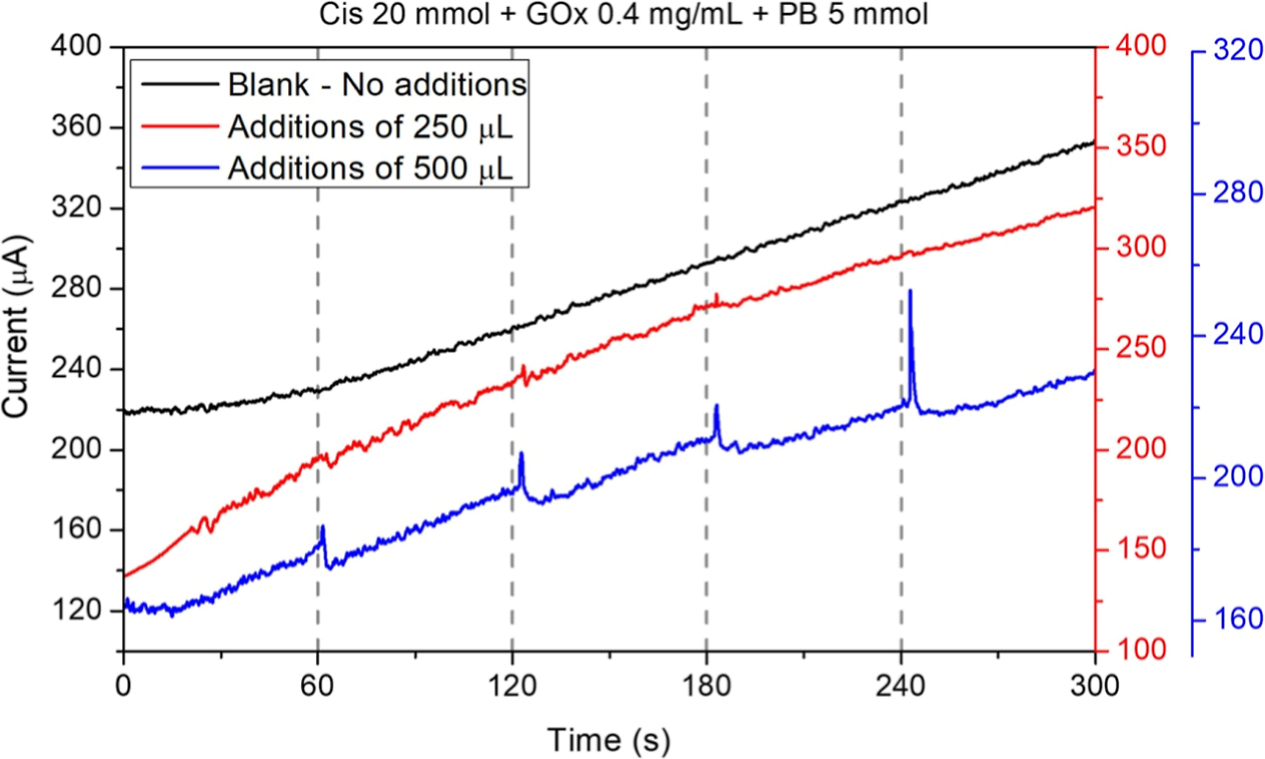
CA response to different added volumes of glucose.

We conducted a comparative study to determine whether a higher concentration of PB could improve the measured current. Biosensors were prepared, and an aliquot of 8 μL from the PB aqueous solution, with 5 mmol (Figure 6A) and 10 mmol (Figure 6B), was dropped over the WE area and dried. As observed, a current variation was detected in all curves after the addition of glucose solutions. Tables 1 and 2 summarize the current variation (Δ*i*) values measured after the addition of glucose aliquots. Sensors fabricated with 5 mM PB consistently exhibited stronger responses across the tested glucose range. The PB acts as a mediator, facilitating electron transfer between glucose oxidase and the electrode. Therefore, optimizing the mediator’s concentration is crucial for biosensor performance. The 5 mM PB-coated electrodes demonstrated superior sensitivity, likely due to optimal mediator loading, which avoided PB agglomeration and enhanced electron transfer, whereas higher PB concentrations (10 mM) led to saturation and diminished current response. Excess PB can form agglomerates or thick films, creating a barrier to electron diffusion and slowing the electrochemical reaction, thereby directly affecting the current response. Using aliquots of 5 mmol, the amount of PB deposited was sufficient to provide a uniform and efficient layer without excess, which could impair electron transfer, resulting in an ideal balance between the amount of mediator and the efficiency of electron transfer, and yielding a better signal than with 10 mmol concentration.

**Figure 6:**
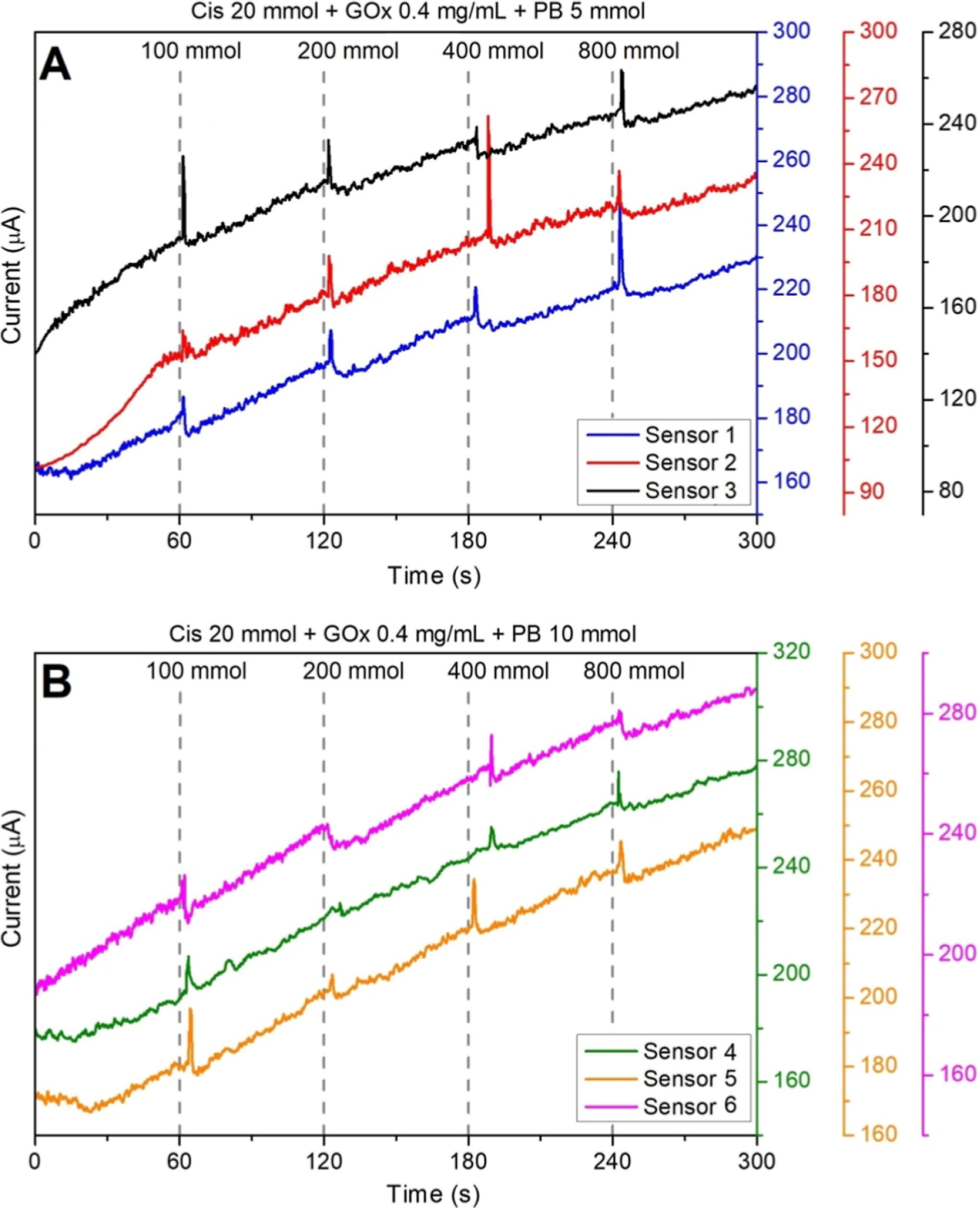
Comparative analysis for enzymatic biosensors decorated with Prussian blue (PB) at (A) 5 and (B) 10 mmol. Additions of glucose solutions every 60 s interval following the concentrations at 100, 200, 400, and 800 mmol.

**Table 1:** Current Variation (Δ*i*) after Addition of Glucose Aliquots in the As-Prepared Biosensor Assembled with PB 5 mmol

	Δ*i* (μA)				
glucose aliquot	sensor 1	sensor 2	sensor 3	SD	RSD (%)
1st addition—100 mmol	10.14	13.88	36.00	13.9	69.8
2nd addition—200 mmol	11.26	15.06	18.60	3.7	24.5
3rd addition—400 mmol	10.20	54.99	9.29	23.9	86.7
4th addition—800 mmol	32.76	17.75	19.94	8.1	34.5

**Table 2:** Current Variation (Δ*i*) after Addition of Glucose Aliquots in the As-Prepared Biosensor Assembled with PB 10 mmol

	Δ*i* (μA)				
glucose aliquot	sensor 4	sensor 5	sensor 6	SD	RSD (%)
1st addition—100 mmol	13.88	16.35	10.75	2.8	20.5
2nd addition—200 mmol	4.03	6.27	6.88	2.5	26.2
3rd addition—400 mmol	8.45	15.01	11.14	3.3	28.6
4th addition—800 mmol	12.21	8.62	7.45	2.5	26.3

Despite a higher standard deviation, the sensors prepared with 5 mmol showed greater current responses to glucose addition (Figure S2). Furthermore, the distribution of Prussian blue was more homogeneous at this concentration, which favors efficient electron transfer and enhances the electrochemical reaction between the glucose oxidase enzyme and the PB layer. In the following experiments, the sensors’ stability was monitored over 8 days, with measurements taken on the third, sixth, and eighth days (Figure 7). During all these days, the sensor was kept immersed in a PBS buffer for evaluation of its long-term performance.

**Figure 7:**
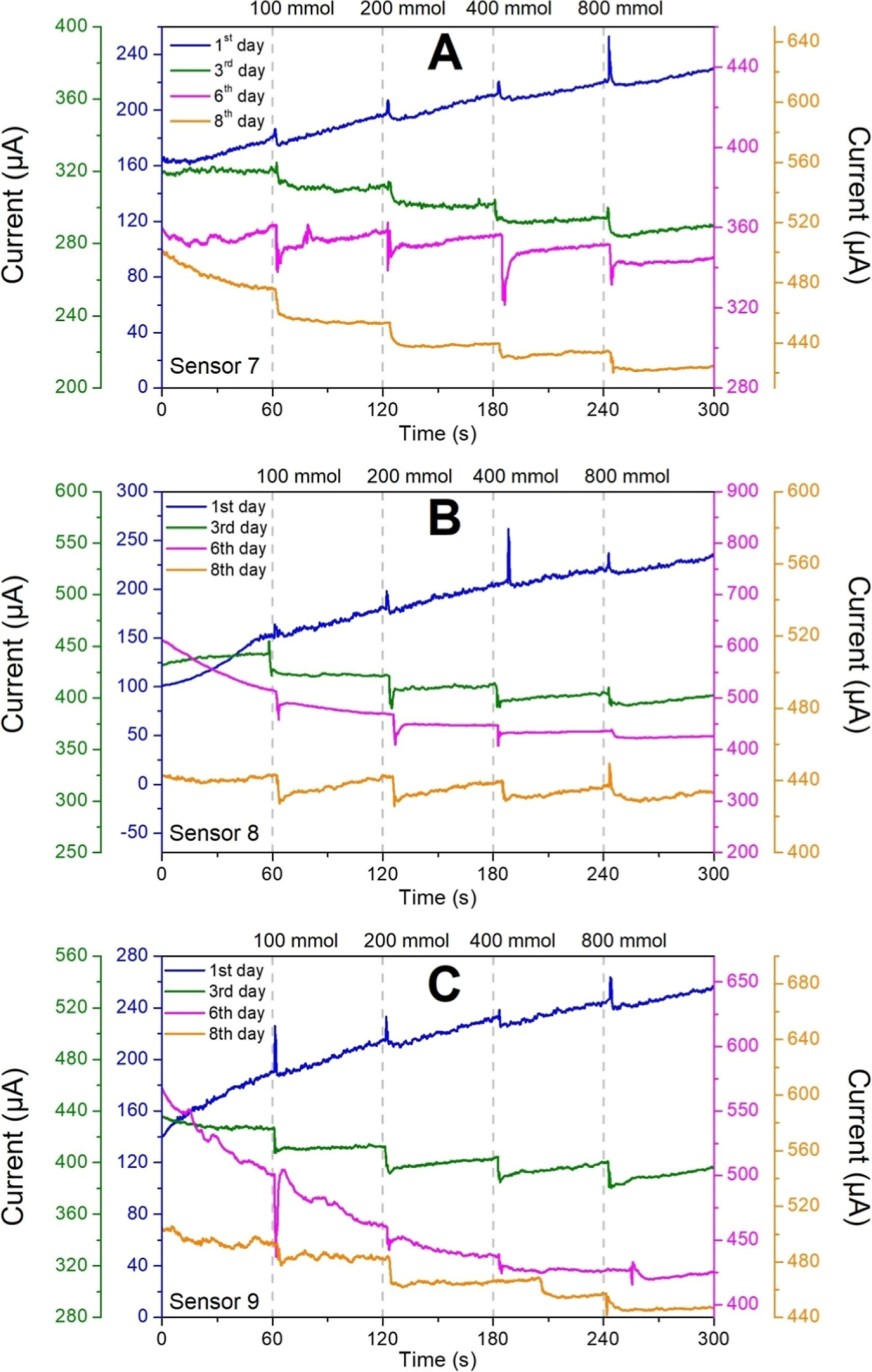
CA response for (A) Sensor 7, (B) Sensor 8, and (C) Sensor 9, prepared with Prussian Blue 5 mmol. Sequential additions at 60 s intervals from glucose aliquots at concentrations of 100, 200, 400, and 800 mmol.

All sensors exhibited a typical current response to sequential additions of glucose at concentrations of 100, 200, 400, and 800 mM. A noticeable change in the baseline behavior was also observed. On the first day (blue line), with news electrodes, the background current increased over time, likely due to gradual equilibration with the electrolyte solution. After 300 s, a current variation of ∼80 μA for Sensor 7, ∼150 μA for Sensor 8, and ∼120 μA for Sensor 9 was observed, with regard to the final and initial measured current. In subsequent days, however, the baseline current decreased, possibly due to the lack of a more efficient washing step between analyses. Since the biosensors were rinsed with fresh PBS to remove residual glucose molecules, this could have facilitated the formation of a thin residual PBS film on the WE surface, altering the electrochemical microenvironment and reducing mass transfer and conductivity. Over time, water evaporation and salt accumulation likely contributed to the formation of an electrolytic layer, partially obstructing the surface and acting as a diffusion barrier, thereby decreasing the baseline current. These findings highlight the importance of implementing washing and regeneration protocols between measurements to ensure the data reliability.

The slight decrease in baseline current observed after the first day can be attributed to the release of weakly adsorbed or partially cross-linked GOx molecules, which are gradually removed during the initial electrochemical cycles. However, greater stabilization of the curves was observed in relation to the final current after the measurements compared with the initial current. Here, for Sensor 7 a variation of ∼30 μA at the third day and at the eighth day was observed; sensor 8 shows a variation of ∼20 μA at the third day and ∼10 μA at the eighth day; and sensor 9 shows a variation of ∼40 μA at the third day and eighth day. This stabilization is attributed to the conditioning of the WE surface, in which adsorbed buffer-solution molecules passivate active sites. This process alters the electrochemical interface, leading to a more predictable and stable response in subsequent measurements.

The CA measurements in Figure 7 provide direct evidence of the sensor’s functionality. Following the addition of glucose aliquots (at 60 s intervals, indicated by the gray dashed line), a swift and reproducible current step was observed, indicating rapid mass transport and catalytic turnover. The summarized data (Table 3–6) show that these step-changes were substantial, consistently producing current variations above 7 μA (also shown in Figure S3), indicating the potentially of our biosensor assembly approach for real-time analyte detection with good sensitivity.

**Table 3:** Current Variation (Δ*i*) in the Biosensor after Glucose Additions on the 1st Day

	Δ*i* (μA)				
glucose aliquot	sensor 7	sensor 8	sensor 9	SD	RSD (%)
1st addition—100 mmol	7.73	12.33	36.29	15.3	81.6
2nd addition—200 mmol	9.63	17.14	14.89	3.8	27.7
3rd addition—400 mmol	9.85	54.15	7.36	26.3	110.6
4th addition—800 mmol	32.48	17.64	18.65	8.3	36.2

**4:** Current Variation (Δ*i*) in the Biosensor after Glucose Additions on the 3rd Day

	Δ*i* (μA)				
glucose aliquot	sensor 7	sensor 8	sensor 9	SD	RSD (%)
1st addition—100 mmol	8.45	20.44	18.31	6.4	40.6
2nd addition—200 mmol	8.41	32.03	20.77	11.8	57.9
3rd addition—400 mmol	8.82	21.22	17.66	6.4	40.1
4th addition—800 mmol	9.96	11.65	19.67	5.2	37.7

**5:** Current Variation (Δ*i*) in the Biosensor after Glucose Additions on the 6th Day

	Δ*i* (μA)				
glucose aliquot	sensor 7	sensor 8	sensor 9	SD	RSD (%)
1st addition—100 mmol	21.01	54.88	62.49	22.1	47.8
2nd addition—200 mmol	13.43	42.40	18.03	15.5	63.2
3rd addition—400 mmol	31.42	39.59	12.60	13.8	49.6
4th addition—800 mmol	19.32	11.93	10.86	4.6	32.8

To assess continuous monitoring capabilities, experiments were conducted with biosensors immersed in PBS containing 70 mg/dL glucose to simulate interstitial fluid analysis. The sensor was kept immersed in the system for 2 days (Figure 8). Sensors remained in this solution for 24 h to achieve electrochemical equilibrium before glucose addition. During the initial 10 min CA analysis (without glucose additions) the background current increased linearly from ∼20 μA to ∼80 μA, likely due to continuous enzyme–electrolyte interactions (Figure 8). Subsequently, five glucose solutions (0.7, 0.8, 0.9, 5, and 11 mg/mL) were prepared and added in 2 mL aliquots to simulate postprandial glucose variations (i.e., blood sugar levels after eating), resulting in final concentrations of 80, 90, 100, 150, and 250 mg/dL in the simulated interstitial system. After each addition cycle, the PBS matrix with 70 mg/dL of glucose was renewed.

**Figure 8:**
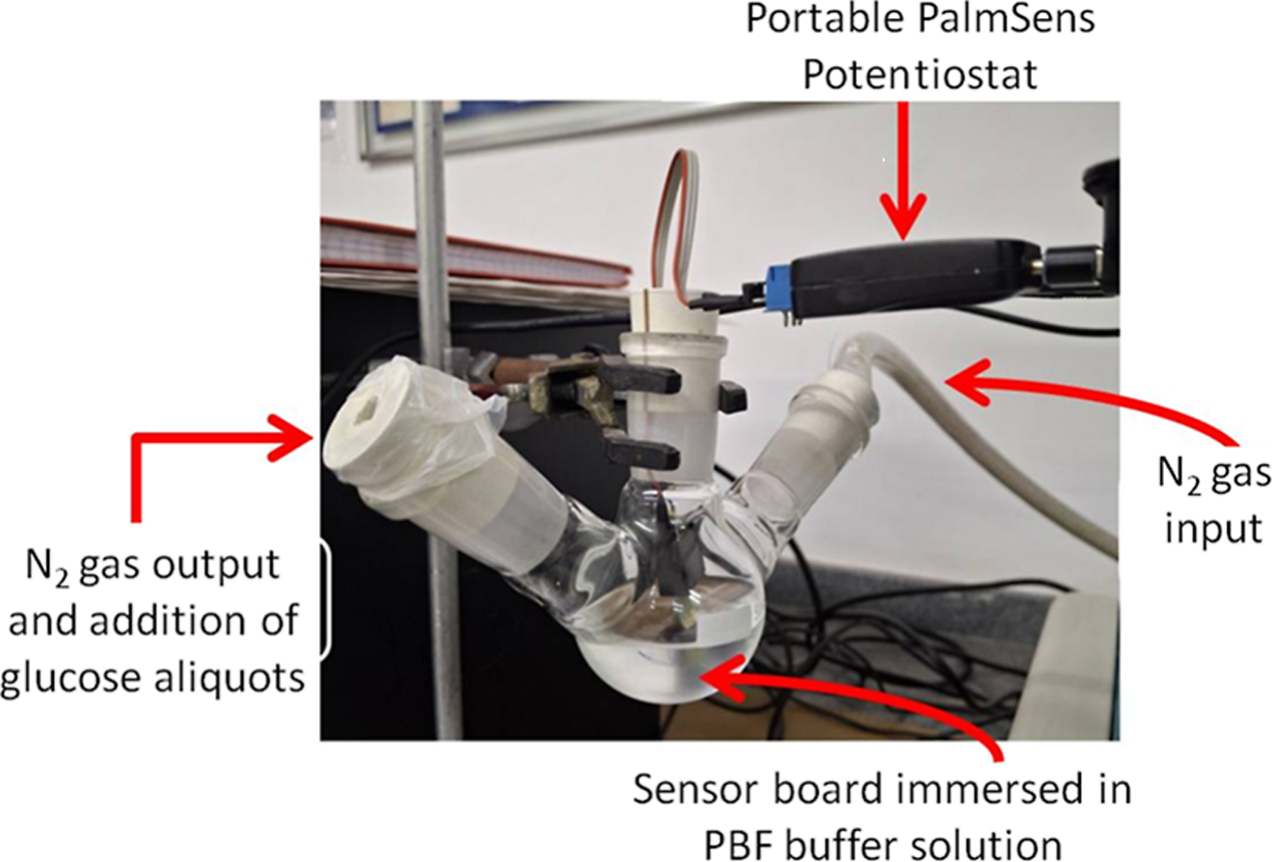
Experimental setup for real-time detection carried out with the enzymatic biosensor immersed in PBS + glucose 70 mg/dL.

**Table 6:** Current Variation (Δ*i*) in the Biosensor after Glucose Additions on the 8th Day

	Δ*i* (μA)				
glucose aliquot	sensor 7	sensor 8	sensor 9	SD	RSD (%)
1st addition—100 mmol	16.08	15.79	15.01	0.5	3.5
2nd addition—200 mmol	14.89	16.19	17.77	1.4	8.8
3rd addition—400 mmol	9.36	10.14	12.26	1.5	14.1
4th addition—800 mmol	14.01	5.32	15.18	5.38	46.8

CA measurements were performed the following day, 24 h after continuous immersion. Analyses conducted in the morning and at the end of the day showed significant amperometric responses to successive glucose additions, with distinct current peaks detected immediately after each addition. The morning measurements (red line) showed peak intensities that were higher than those of the afternoon measurements (blue line), suggesting a slight decline in enzyme activity over time.

As shown in Figure 9, the biosensor maintained its ability to respond to glucose additions even after extended immersion, indicating that the catalytic activity of glucose oxidase and the electron mediation of Prussian Blue remained intact during continuous operation. This persistence demonstrates that combining cystamine SAMs, glutaraldehyde cross-linking, and an optimized PB layer creates a robust biorecognition interface. The interface withstands prolonged exposure to aqueous media. Such behavior underscores the sensor’s potential for CGM platforms, where durability and reproducibility are essential. While the proof-of-concept validates stability over several hours, future adaptations would be necessary. These could include adding protective polymer membranes, antifouling surface modifications, or integrating microfluidic sampling systems to ensure biocompatibility and extend the lifespan to clinically relevant time scales.

**Figure 9:**
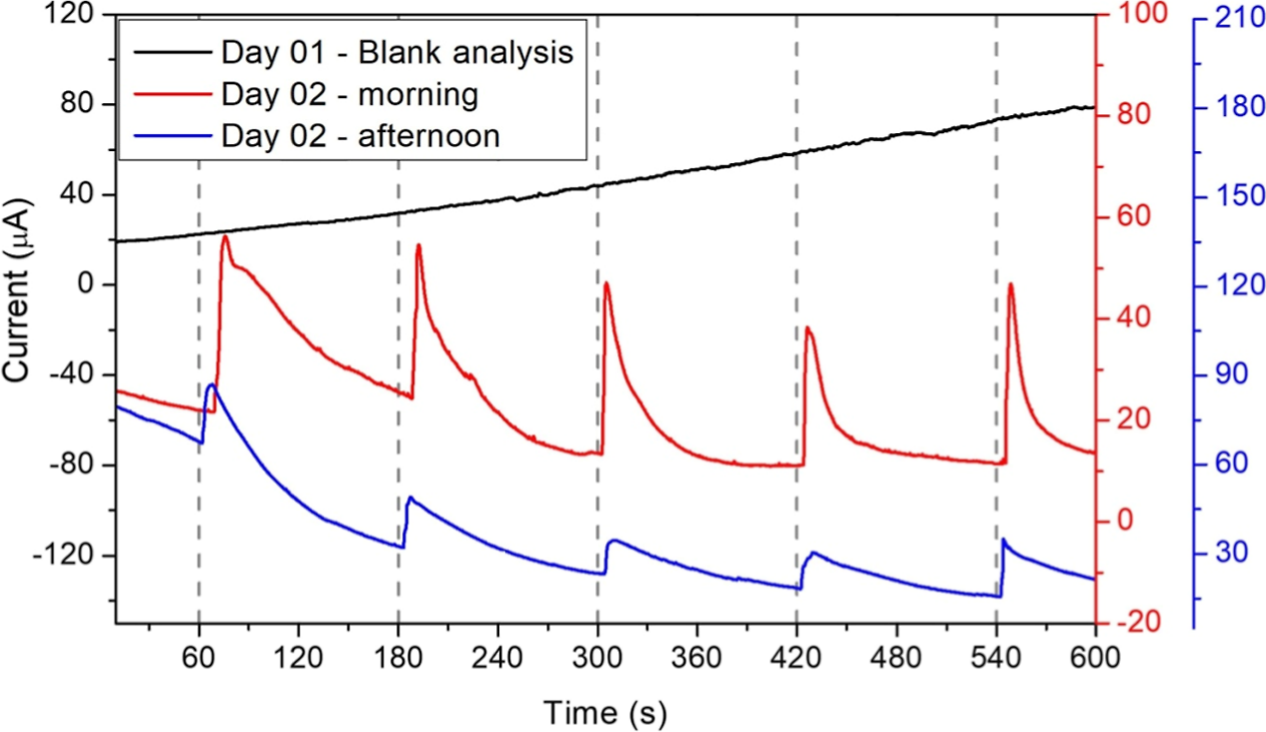
CA response for the sensor immersed in a matrix solution of PBS + glucose 70 mg/dL.

Table 7 details the current variation (Δ*i*) for each glucose aliquot addition. The average peak height during the first morning measurements was approximately 32 μA, while the average peak height during the afternoon measurements was ∼15.8 μA. In this immersed configuration, current peaks of similar intensity were observed with low standard deviation (SD), as shown in Figure S4. These results indicate that the biosensor has improved the sensitivity and did not exhibit saturation over the immersion period, supporting its potential for continuous glucose monitoring applications. In the continuous monitoring simulation (PBS + 70 mg/dL glucose), the sensor showed a higher current response in the morning than in the afternoon after 24 h of immersion, indicating a gradual loss of activity over time. This behavior suggests that the operational lifespan of the biosensor under continuous use is approximately 1 day. The decline in signal can be attributed to progressive deactivation of glucose oxidase (GOx), driven by conformational changes and the partial leaching of enzyme molecules that are not fully stabilized within the cystamine-glutaraldehyde matrix. Moreover, continuous electrocatalytic turnover of hydrogen peroxide may generate reactive species that induce local oxidative stress, further accelerating enzymatic degradation. Similar trends have been reported in continuous biosensing systems, where signal drift and performance decline are common challenges associated with long-term operation.^74,75^ Collectively, these results emphasize the need for protective strategies, such as polymer encapsulation or antioxidant coimmobilization, to extend sensor durability in future developments.

**Table 7:** Current Variation (Δ*i*) in the Biosensor (Immersed in Simulated Interstitial Fluid) after Glucose Additions

enzymatic biosensor	glucose aliquot	Δ*i* (μA)	SD	RSD (%)
day 02 morning	1st addition	34.49	3.65	11.4
	2nd addition	30.29		
	3rd addition	33.71		
	4th addition	26.49		
	5th addition	35.34		
day 02 afternoon	1st addition	19.83	3.76	23.7
	2nd addition	16.91		
	3rd addition	11.41		
	4th addition	12.38		
	5th addition	18.70		

## 4 Conclusions

In this study, we successfully developed a PCB-based electrochemical enzymatic biosensor for glucose detection. The device integrates a cystamine-based SAM on an ENIG-finished PCB substrate, enabling stable, well-oriented immobilization of glucose oxidase (GOx). In addition, a nanostructured Prussian Blue (PB) layer, optimized at 5 mM, was incorporated as an efficient redox mediator to enhance the electrochemical response. The cystamine SAM played a dual role, improving enzyme immobilization and directing homogeneous deposition of PB. This enhanced electron transfer leads to reproducible CA responses. The SAM configuration on the ENIG-finished PCB allowed for immediate, measurable current changes across a broad glucose range. Stability assays showed consistent performance over several days, though baseline drift highlighted the need for washing and regeneration protocols. In simulated interstitial fluid, continuous monitoring tests showed that the biosensor remained responsive even after prolonged immersion, underscoring the robustness of the SAM-PB-enzyme design on ENIG-finished PCBs for CGM applications.

To advance practical use as a subcutaneous CGM on scalable ENIG-finished PCB-based platforms, it is critical to integrate optimized microneedle fabrication strategies. In addition, conducting comprehensive reliability assessments and extended in vivo validation studies is also needed to confirm long-term functional stability, sustained electrochemical performance, and biocompatibility.

## Supplementary Material



## Notes

Biosensor schematic assembly; current variation in sensors with different concentrations of Prussian Blue; current variation in the sensor during a 8 days of analysis; and a biosensor immersed in a simulated interstitial fluid (PDF)
