# Supplementary material for: Layered Enzymatic Biosensor Decorated with Prussian Blue Structures for Continuous Electrochemical Glucose Sensing
Source: ACS Omega. 2026 Apr 16;11(16):23795–807. doi: 10.1021/acsomega.5c08879 (PMC13129871; doi:10.1021/acsomega.5c08879)
Supplement: Supplementary file 1 [file ao5c08879_si_001.pdf]

SUPPORTING INFORMATION

Layered Enzymatic Biosensor Decorated with Prussian Blue Structures for Continuous Electrochemical Glucose Sensing

Aline Macedo Faria[1*], Glauco Meireles Mascarenhas Morandi Lustosa[1,*], Talita Mazon[1*]

[1]Centro de Tecnologia da Informação Renato Archer, Ministério da Ciência, Tecnologia e Inovação (MCTI), Campinas/SP, Brazil

*The authors contributed equally

***Corresponding authors**

Aline Macedo Faria
Centro de Tecnologia da Informação Renato Archer,
Ministério da Ciência, Tecnologia e Inovação (MCTI)
Dom Pedro I Highway, Km 143.6, 13069-901
Campinas/SP, Brazil
e-mail address: aline.faria@cti.gov.br

Glauco Meireles Mascarenhas Morandi Lustosa
Centro de Tecnologia da Informação Renato Archer,
Ministério da Ciência, Tecnologia e Inovação (MCTI)
Dom Pedro I Highway, Km 143.6, 13069-901
Campinas/SP, Brazil
e-mail address: glauco.lustosa@cti.gov.br

Talita Mazon
Centro de Tecnologia da Informação Renato Archer,
Ministério da Ciência, Tecnologia e Inovação (MCTI)
Dom Pedro I Highway, Km 143.6, 13069-901
Campinas/SP, Brazil
e-mail address: talita.mazon@cti.gov.br

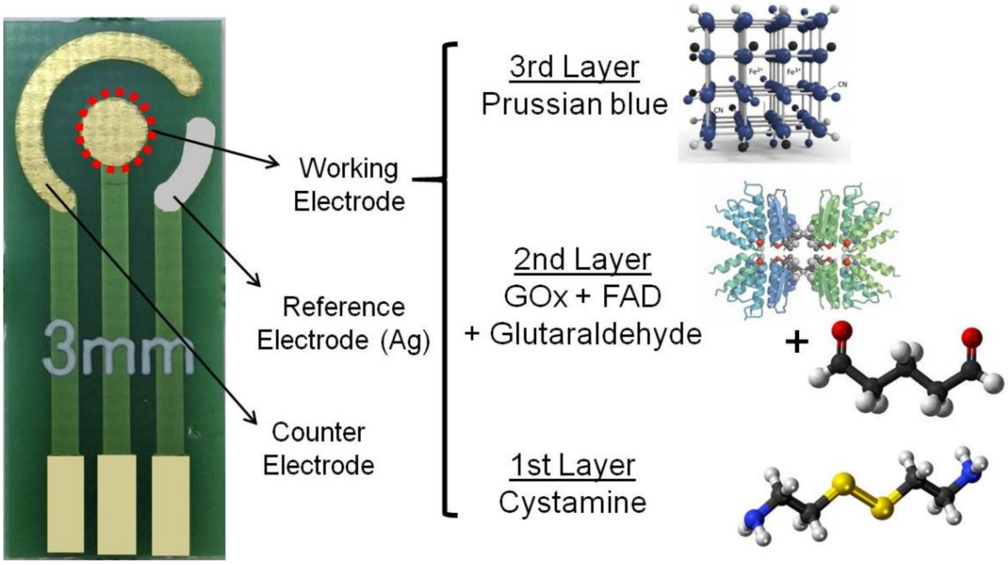

Figure S1 – Schematic representation of the multilayer configuration on working electrode for our biosensor for glucose real-time monitoring.

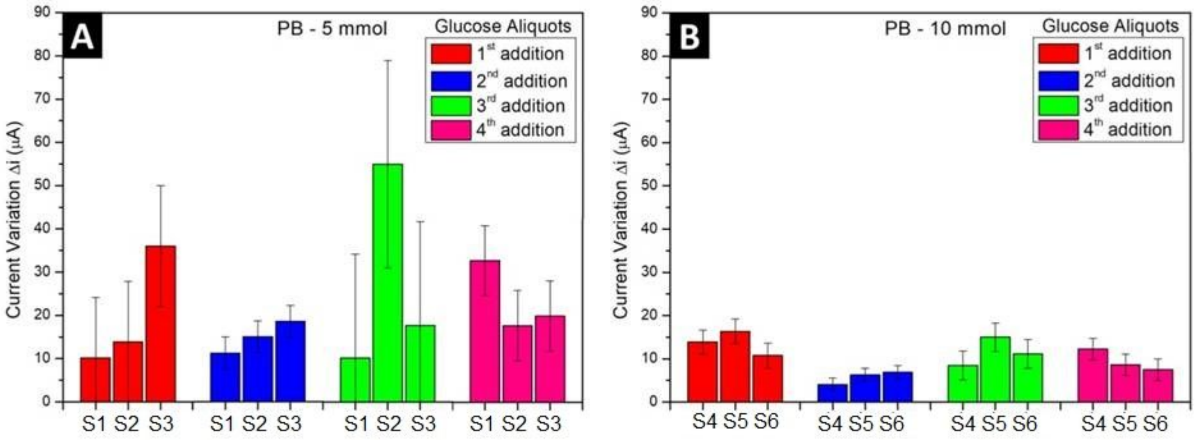

Figure S2 – Current variations (Δi) in the biosensors assembled with Prussian blue 5 and 10 mmol.

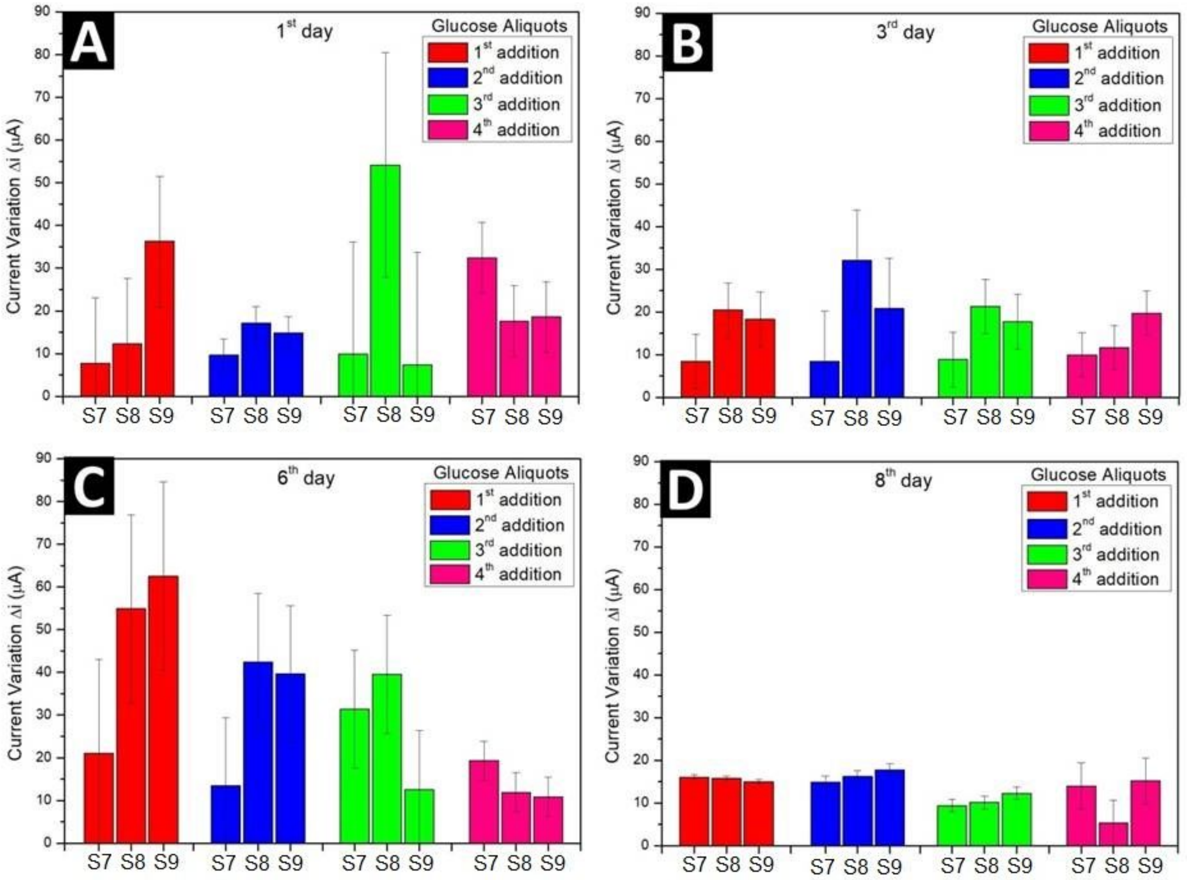

Figure S3 – Current variations (Δi) in the biosensors assembled with Prussian blue 5 mmol through 8-days analysis.

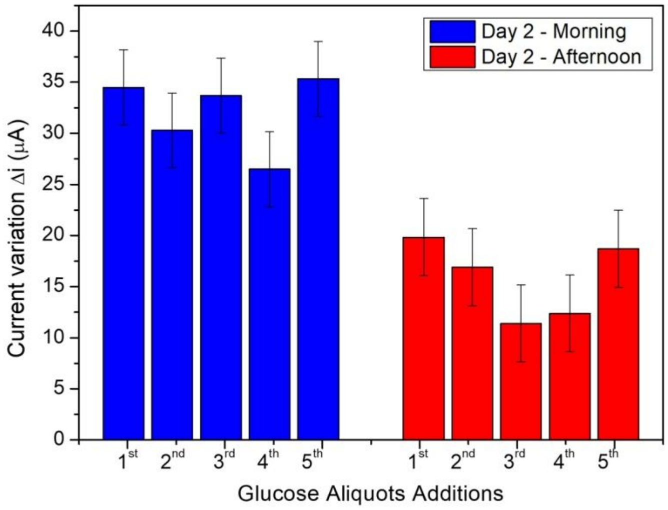

Figure S4 – Current variations (Δi) in the biosensor immersed in a simulated interstitial fluid during 48 hours.
